# Notochordal conditioned media from tissue increases proteoglycan accumulation and promotes a healthy nucleus pulposus phenotype in human mesenchymal stem cells

**DOI:** 10.1186/ar3344

**Published:** 2011-05-31

**Authors:** Devina Purmessur, Rachel M Schek, Rosalyn D Abbott, Bryan A Ballif, Karolyn E Godburn, James C Iatridis

**Affiliations:** 1 Leni and Peter W. May Department of Orthopaedics, Mount Sinai School of Medicine, One Gustave L. Levy Place, Box 1188, New York, NY 10029-6574, USA; 2 The University of Vermont, 33 Colchester Avenue, Burlington, VT 05401, USA; 3 Department of Biology and Vermont Genetics Network Proteomics Facility, The University of Vermont, 109 Carrigan Drive, Burlington, VT 05405, USA

## Abstract

**Introduction:**

Notochordal cells (NCs) are influential in development of the intervertebral disc (IVD) and species that retain NCs do not degenerate. IVD repair using bone marrow derived mesenchymal stem cells (MSCs) is an attractive approach and the harsh microenvironment of the IVD suggests pre-differentiation is a necessary first step. The goal of this study was to use soluble factors from NCs in alginate and NCs in their native tissue to differentiate human MSCs to a young nucleus pulposus (NP) phenotype.

**Methods:**

Human MSCs (cultured under micromass conditions for 21 days in hypoxia) were differentiated with conditioned medium derived from porcine notochordal cells in native tissue (NCT) or in alginate beads (NCA), and compared with chondrogenic (TGFβ-3) or basal medium. A PCR array of 42 genes was utilized to screen a large number of genes known to be associated with the healthy NP phenotype and pellet cultures were also evaluated for glycosaminoglycan content, histology and viability. Proteomic analysis was used to assess candidate soluble factors in NCA and NCT.

**Results:**

Notochordal cell conditioned media had diverse effects on MSC phenotype. NCT resulted in the highest levels of glycosaminoglycan (GAG), as well as up-regulation of SOX9 and Collagen II gene expression. NCA demonstrated effects that were catabolic yet also anti-fibrotic and minimally hypertrophic with down-regulation of Collagens I and III and low levels of Collagen X, respectively. Micromass culture and hypoxic conditions were sufficient to promote chondrogenesis demonstrating that both basal and chondrogenic media produced similar phenotypes. Candidate matricellular proteins, clusterin and tenascin were identified by proteomics in the NCA group.

**Conclusions:**

NCs secreted important soluble factors capable of differentiating MSCs to a NP phenotype synthesizing high levels of proteoglycan while also resisting collagen fiber expression and hypertrophy, yet results were sensitive to the conditions in which media was generated (cells in alginate versus cells in their native tissue) so that further mechanistic studies optimizing culture conditions and defining important NC secreted factors are required. Matricellular proteins, such as clusterin and tenascin, are likely to be important to optimize differentiation of MSCs for maximum GAG production and reduced collagen fiber expression.

## Introduction

Current surgical therapies to treat intervertebral disc (IVD) degeneration include spinal fusion and arthroplasty; these methods are highly invasive and are often associated with reduced patient mobility [1]. Cell based therapies are an attractive alternative since they may be applied in a minimally invasive manner with the ability to address an underlying cause of degeneration. IVD degeneration is associated with increased cell apoptosis and senescence, an up-regulation of pro-inflammatory and pain-related proteins, and ultimately, a breakdown of the disc matrix [2-5]. Cell-based therapies aim to restore metabolic homeostasis within the IVD and reduce inflammation by replacing or augmenting the disc cells at an early stage of degeneration. Such therapies can adapt and integrate with the native tissue microenvironment restoring structure and function with limited long term side effects. One promising cell choice is mesenchymal stem cells (MSCs). MSCs are multipotent cells predominantly found in bone marrow that have the plasticity to differentiate into cells of the chondrocytic, adipogenic and osteogenic lineages [6]. However, there is evidence to suggest that MSCs may not be well suited to the hostile anaerobic environment of the diseased IVD [7,8] so that long term survival and integration within the disc may require pre-differentiation of the MSCs in culture towards a phenotype more representative of native IVD cells.

There are at least two cell populations in the disc, the fibrochondrocytes that populate and maintain the annulus fibrosus (AF) and the more chondrocytic cells in the nucleus pulposus (NP). The NP cells are often described as being "chondrocyte-like" as a consequence of their morphology and the extracellular matrix proteins they synthesize (such as collagen type II and aggrecan). The glycosaminoglycan (GAG) to hydroxyproline ratio is an important distinguishing characteristic between NP cells with ratios as high as 27:1 and hyaline chondrocytes with ratios as low as 2:1 [9].

MSCs are a promising potential cell source for IVD repair, as described by a number of *in vitro *and *in vivo *studies [10-19]. The interaction between MSCs and cells of the native IVD, including the adaptation of MSCs to the IVD microenvironment, enhanced MSC metabolism and biosynthesis; however, the magnitude of effects appears to be dependent on cell ratio and whether the cell contact is indirect or direct [12,18-20]. Studies suggest that a ratio of 75% NP:25% MSC with direct cell-cell contact provides the optimal culture conditions for MSC differentiation and matrix expression toward a chondrocyte-like phenotype [18]. This interaction appears to be independent on MSC source, as both autologous and allogenic MSCs interact favorably with NP cells [16,19]. *In vivo*, the ability of MSCs to improve biosynthesis and restore homeostasis within degenerated IVD is likely to be dependent on their long term survival in the native IVD microenvironment. Injection of undifferentiated MSCs into the IVDs of small animal models such as degenerated rabbit IVDs depleted of NP tissue demonstrated survival of MSCs for up to 48 weeks [14]. However, the tissue composition (NP matrix) and cell populations (predominantly notochordal NP cells) in these animal models differ radically from those present clinically in human degenerated IVDs.

Differentiation of MSCs toward an NP phenotype is more complex than differentiation towards a hyaline chondrocyte lineage [21]. Differentiation toward an NP phenotype is likely to depend on diverse biological parameters such as an appropriate choice or combination of growth factors, 3D matrix, cell-cell contact and environmental conditions mimicking the IVD such as hypoxia. Further, only very recently, has the phenotype of NP cells become more clearly defined. While no single definitive NP marker exists, many laboratories have examined potential markers associated with a "healthy NP phenotype" in a diverse range of animal species including human IVDs and these studies are ongoing [22-26]. The proteoglycan-rich matrix and high proteoglycan to collagen ratio of the human NP is considered an important marker when determining a healthy NP phenotype [9]. Based on literature a healthy NP like phenotype can be considered as high proteoglycan biosynthesis, increases in the matrix proteins SOX9, collagen II, aggrecan, phenotypic markers such as keratin-19 and transforming growth factors 1 and 3 (TGFβ-1 and -3) [10,22,27,28]. This is coupled with decreases in collagens I, III and X, the catabolic enzymes matrix metalloproteinases (MMPs) and A distintegrin and metalloproteinase with thrombospondin motifs (ADAMTSs) and inflammatory cytokines interleukin-1β (IL-1 β) and tumour necrosis factor alpha (TNFα) [2,4,29,30],

The notochord plays an influential role in early development of the IVD [31] and exposing MSCs to notochordal cells (NCs) has been proposed as a powerful method for differentiation to an NP phenotype [32]. In a number of species, including humans, during growth and aging, the NCs populating the NP disappear and are replaced by chondrocytic NP cells [33]. The NP of some species retain notochord cells into maturity, for example, the pig, rabbit, non-chondrodystrophoid dogs and rodents, and the IVDs of these species do not experience degeneration of the IVD [33], suggesting an association between NCs with IVD development and maintenance of the healthy NP phenotype. It has previously been shown that NCs, including conditioned medium derived from NCs (NCCM) has enhanced IVD cell and articular chondrocyte metabolism [34,35]. More recent studies by Korecki *et al*. have shown that NCCM from porcine NCs seeded in alginate increased GAG production and up-regulated Laminin B1 and collagen type III in human MSCs after seven days in culture [32]. While NCCM demonstrates strong promise for NP differentiation, generation of NCCM was not optimized. For example, Korecki *et al*. employed NCs isolated from their native tissue environment in order to highlight the relevance of NCs alone. Yet, the cell matrix interactions will influence the production of soluble factors from NCs which maintain the healthy IVD, and it is speculated that generation of NCCM within the native tissue environment has anabolic soluble factors that may improve differentiating potential of MSCs cells to an NP phenotype.

We hypothesize that NCCM generated from NCs in their native tissue environment will trigger differentiation of MSCs toward an NP phenotype to a greater extent than both notochordal media generated from NC cells in alginate and chondrogenic media (TGFβ-3) alone. The first aim of this study was to pre-differentiate MSCs into cells with a healthy NP-phenotype based on custom PCR array analysis and GAG production as defined above. A custom PCR array was designed to evaluate expression of 42 genes chosen from recent literature in order to characterize NP cell phenotype, matrix protein, catabolic/anti-catabolic protein, growth factor and pain/inflammatory protein expression. The second aim was to identify the optimal conditions for generating conditioned media by comparing the effects of CM derived from NCs seeded in alginate or derived from notochordal tissue, as compared with chondrogenic media with TGFβ-3. The last aim consisted of a pilot study of proteomic analysis of secreted protein factors from the NCT and NCA conditioned media that may provide instructive cues and create unique extracellular environments that would contribute to our understanding of how NCs influence development of a healthy NP phenotype.

## Materials and methods

### Generation of conditioned media from porcine IVD cells and tissue

The average ratio of notochordal to NP cells isolated from the IVDs of each pig spine was 88%:12%, similar to that found by Chen *et al*. [36]; therefore, the whole pool of NP cells were taken to be predominantly notochordal in nature. NP tissue was carefully isolated aseptically from IVDs of two- to eight-month-old female porcine spines (*n *= 8) obtained within 24 hours of death (Animal Facility Research 87 Inc., Boylston, MA, USA). To generate conditioned media (CM) from notochordal cells seeded in alginate beads (NCA), NP tissue was first digested as described by Urban *et al*. [37]. Briefly, tissue was digested with 0.2% protease (from *Streptomyces griseus *- Type XIV: P5147, Sigma-Aldrich, ST Louis, MO USA) for one hour followed by 0.025% collagenase (from *Clostridium histolyticum *type 1A: C2674, Sigma-Aldrich) for 18 hours at room temperature. To remove remaining cell clusters, additional digestion with Cell dissociation solution, non-enzymatic 1 × (C1419, Sigma-Aldrich) was performed for two hours. Cells were then rinsed in 0.15 M NaCl and encapsulated in beads at a density of 2 × 10^6 ^cells/ml of 1.2% low viscosity alginate (Sigma-Aldrich). Beads were cultured in 12-well plates at a density of 10 beads/well with 2 ml of media (low glucose DMEM, 1% Pen/Strep, 0.5% Fungizone and 1% insulin-transferrin-selenium (ITS) (I2771, Sigma Aldrich)) for four days in hypoxia (5% O_2_, 5% CO_2_, 37°C).

For generation of CM from notochordal cells in tissue (NCT), the NP of three porcine discs (wet weight approximately 0.9 to 1.3 g) were soaked per 30 mls of low glucose DMEM, 0.5% Fungizone and 1% Pen/Strep without ITS for four days in hypoxia. Media was retained and filtered through a 70 um cell strainer (Thermo Fisher Scientific, Pittsburg, PA USA) to remove any remaining tissue.

NCA and NCT were both filtered through MW 3000 Amicon Ultra-15 (Millipore Bedford, MA USA) and re-suspended in 15 ml Basal media (B) (low glucose DMEM + 1% Pen/Strep + 1% ITS) in order to remove small metabolites and waste products. 15 ml of either NCT or NCA was added to each Amicon Ultra-15 filter and material on top (the concentrate) was resuspended in 15 ml Basal media with a final concentration of 1 ×. To verify the conditioned media used was the same from each notochordal culture all media was pooled for NCT and NCA respectively.

### Pelleting of MSCs

Human bone marrow derived MSCs samples (age range 22 to 37 years, *n *= 3) were purchased from Texas A&M (Temple, TX, USA) with the appropriate Material Transfer Agreement and expanded in monolayer culture in alpha MEM medium supplemented with 10% fetal bovine serum. At passage 4, cells were pelleted at a density of 250,000 cells in 15 ml polypropylene tubes by centrifugation at 600 g for five minutes. They were then cultured in 500 μl of Chondrogenic media (C) (Basal media with 50 μg/ml ascorbate, 0.1 μM dexamethasone, 40 μg L-proline and 10 ng/ml TGFβ-3) in hypoxic conditions for 24 hours.

### Treatment of MSCs with 4 CM types: B, NCT, NCA, and C

After 24 hours, spent media was removed and 500 μL of either B, NCA, NCT or C were added to respective tubes containing pellets (Table 1: study design) and changed every three to four days, for a total culture period of 21 days. Media was retained to assess GAG released to media during the 21-day culture.

**Table 1:** Study design for treatment of MSCs with B, C, NCA and NCT groups

Media group	Number of human samples	Time in culture	Number of pellets per sample			
			PCR	GAG	Cell viability	Alcian blue stain
B	3	21 days	5	3	1	2
C	3	21 days	5	3	1	2
NCA	3	21 days	5	3	1	2
NCT	3	21 days	5	3	1	2

### Dependent variables

#### PCR array

Each pellet was lysed with 300 μl RNeasy Lysis RLT buffer (Qiagen: 79216 Valencia, CA USA) and the lysates from five pellets pooled stored at -80°C. RNA was extracted, cDNA synthesized and custom RT^2 ^Profiler' SYBR green PCR arrays (SABiosciences: CAPH-0817A (Qiagen), Frederick, MA USA) were run by the Vermont Cancer Centre DNA analysis facility. The custom array included 42 genes associated with NP cell function: Phenotypic marker/Matrix-associated protein genes, growth factor genes, catabolic/anti-catabolic genes and inflammatory/pain genes (Additional file 1 Table S1 and Additional file 2 Table S2). Relative gene expression was calculated using the comparative Ct method normalized to undifferentiated MSCs from the same patients (Day 0) and three housekeeping genes (18s, GAPDH and β-actin). For normalization purposes, undetermined values for Day 0 were given an arbitrary value of 40 as undifferentiated MSCs did not express all the genes (leading to some catabolic genes with artificially high fold increases). Error bars were plotted as SEMs.

#### Glycosaminoglycan (GAG) and DNA content

To examine GAG and DNA in the cell pellet, spent media was removed and 200 μl of lysis buffer (Sigma Aldrich: L-8285; RNT70) was added to each cell pellet. This lysis buffer is routinely used to lyse cell membranes for the release of RNA/DNA and was also used to dissociate GAG associated with the cell pellet. The lysate was then assessed using the Di-methyl methylene Blue (DMMB) assay and the standard curve was generated in the lysis buffer used to dissociate the cell pellets. DMMB was then normalized to DNA content using the picogreen assay (Invitrogen, Carlsbad, CA, USA). To quantify the GAG released to media, media samples from the pellets of each media group retained over 21 days were assessed and each GAG measurement subtracted from the respective Day 0 control media (NCA. NCT, C and B before addition to pellets) averaged for the total 21 days and then normalized to DNA content [38].

#### Cell viability

Viability was analysed with the Live/Dead Kit (Invitrogen). Briefly, media was removed and the pellets were washed with PBS. Each pellet was resuspended in 100 μl of a 2 μM Calcein AM/1 μM Ethidium Homodimer-2 (ETH-2) staining solution and the cell suspension placed onto a microscope slide. Cells were incubated in the dark at 37°C for 30 minutes. After incubation a cover-slip was placed on top of the suspension and cells were visualized at 20 ×. Excitation and emission for Calcein and ETH-2 were 494/517 nm and 528/617 nm respectively with Calcein staining the cytoplasm of live cells green and ETH-2 staining the nuclear envelope of dead cells red.

#### Histology of pellets

To visualize the intact pellet, pellets were first fixed in formalin and then incubated with 1% Alcian Blue in HCl for 30 minutes, followed by final fixation in 100% ethanol. Pellets were embedded in freezing medium and 20 μm sections cut using a cryotome. Sections were re-stained with 1% Alcian Blue in HCL for 30 minutes in order to ensure full penetration of the dye to assess proteoglycan quantity and location, and also 4',6-diamidino-2-phenylindole (DAPI, Roche Diagnostics, Mannheim, Germany) which stains the nuclei of cells (Ultraviolet filter), followed by a wash with PBS. Images were captured on an Olympus BX50 light microscope (Center Valley, PA USA) at 20 × magnification.

### Mass spectrometry and data analysis

While the presence of significant amounts of albumin (present in the ITS solution) greatly reduced the signal to noise and may have masked several important proteins, distinct bands running at approximately 37 kDa and 140 kDa for the NCT and NCA groups were observed (Additional file 3 Figure S3). Gel regions corresponding to these molecular weights were excised from each of the three lanes and subjected to in-gel tryptic digestion. Coomasie-stained regions of the SDS-PAGE gel were diced into 1 mm cubes. Gel pieces were reduced, alkylated with iodoacetamide and subjected to in-gel digestion as described previously [39]. Dried peptides were subjected to liquid chromatography tandem mass spectrometry in a linear ion trap (LTQ) mass spectrometer (Thermo Electron Corporation Waltham, MA USA). Data were searched against the International Protein Index (IPI) non-redundant protein database using Sequest; requiring tryptic specificity; allowing precursor m/z tolerances of 2 Da; allowing methionine residues to be oxidized (+15.99 Da); and requiring cysteine residues to be carbamidomethylated (+57.02 Da). Peptides were filtered initially by requiring a XCorr value > 2 for doubly-charged peptides and > 2.5 for triply-charged peptides. Proteins having more than three peptides meeting these criteria were retained and XCorr values were then relaxed for peptides from these proteins to > 1.7 for doubly-charged peptides and > 2 for triply-charged peptides. Proteins that were common to unconditioned and conditioned media were discarded as well as proteins that did not have at least two porcine-specific peptides or had peptide that were not consistent with porcine origin as determined by the SEQUEST search analysis and manual BLAST analysis of each remaining peptide. To estimate the peptide false-discovery rate for the peptides identified in this study, we employed a statistical method using a target-decoy strategy as described in detail previously [40] As the complete porcine proteome is not available, and as the IPI indexed non-redundant database is not formatted for the generation of a decoy database, we searched all MS data against a concatenated forward (target) and reverse (decoy) IPI human protein database containing the sequences of the proteins harboring the porcine-specific proteins identified in this study. Using the same filtering criteria used for the non-redundant database search, we filtered the data to a false discovery rate of less than 0.01% at the peptide level. The proteins harboring porcine-specific peptides were again identified and remained in this dataset after filtering. Thus, the odds that any one of the identified peptides from these proteins is a false positive are less than 0.01%.

### Statistics

For qRT-PCR, statistical analysis was performed first by testing for normality using a Ryan-joiners test. For samples that were either parametric or non-parametric, a one sample t-test or one sample sign rank test of the ΔΔCt values with hypothesized value of 0 were carried out respectively (ΔΔCt for B, C, NCA and NCT groups and ΔΔCt = 0 for undifferentiated MSCs at Day 0/or B). GAG was assessed for normality and a one-way ANOVA with a Fisher's PLSD was done in order to check for significance between all media treatment groups (with *P *< 0.05 significant). Similar analysis using a one-way ANOVA with a Fisher's PLSD was carried out for DNA content. For a description of the statistical approach used in the proteomic analysis see the mass spectrometry and data analysis section above.

## Results

### Gene expression data (significance > 2-fold normalized to Day 0 and B)

Gene expression data was normalized to Day 0 undifferentiated MSCs and also to B conditioned MSCs because of similarities between B and C groups. Only gene expression data with significance greater than two fold was discussed.

#### Phenotypic marker genes

**Figure 1:**
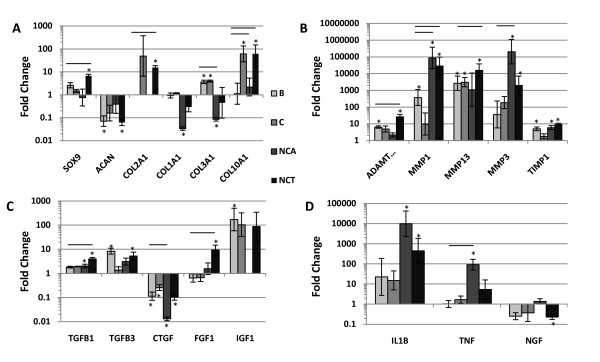
**Gene expression data in MSCs 3 weeks post treatment**. qRT-PCR results for human mesenchymal stem cells (MSCs) (*n *= 3) pelleted in 3D culture and treated with conditioned media from four groups: B = Basal, C = Chondrogenic, NCA = notochordal NP cells in alginate; NCT = notochordal NP cells in tissue for 21 days. Fold change in mRNA levels were calculated with the ΔΔC_T _method relative to three housekeeping genes and undifferentiated (Day 0) MSCs from the same patient. * indicates significantly different from Day 0 and bar indicates significantly different from Basal, *P *< 0.05; Error bars are expressed as SEMs. **1A: **Phenotypic marker/Matrix-associated protein genes. The most prominent results were the significant increase in Sox9 and Col2A1 for NCT media compared to basal and the significant decrease in Col 1A1 and Col3A1 for NCA media; **B: **Catabolic/anti-catabolic genes. All media conditions had high expression levels for catabolic genes. Very low levels of mRNA for catabolic proteins resulted in very high relative expression levels relative to Day 0 controls. Most relevant comparisons, therefore, are with other groups; **C: **Growth factor genes. A general up-regulation in expression was observed in particular for NCT, with the exception of CTGF with all media groups; **D: **Inflammatory/pain genes. Up-regulation of pro-inflammtory cytokines was noted for NCA and NCT however significant down-regulation of NGF was observed with NCT.

Treatment of MSCs with B, C, NCA and NCT for 21 days in pellet culture had diverse effects on the gene expression of phenotypic markers with few differences observed compared to the basal group for these genes (Table 2). C demonstrated no significant changes compared to Day 0 or B for any IVD markers. B demonstrated a significant increase in the gene expression of the IVD marker *GPC1 *compared to Day 0. NCA showed a significant down-regulation of *BGN *relative to Day 0 and B. Only NCT demonstrated significant up-regulation of *SOX9 *relative to Day 0 and B (Figure 1A), although a significant down-regulation of *KRT19 *relative to Day 0. A trend of up-regulation of adipogenic (*PPARG*) and osteogenic (*BGLAP*) markers was observed for all treatment conditions.

**Table 2:** Fold changes in gene expression data for media conditions B, C, NCA and NCT Significance greater than two-fold relative to Day 0 MSCs or Basal in bold and underlined; + = fold increase & - = fold decrease (*P *< 0

ALL GENES			Normalized to Day 0				Normalized to Basal		
			B	C	NCA	NCT	C	NCA	NCT
		BGN	-1.0	+1.1	**-6.1**	+1.6	+1.1	**-6.2**	+1.6
		GPC1	**+2.5**	+2.1	+1.5	+1.9	-1.2	-1.7	-1.2
	**IVD**	KRT19	-7.3	-1.0	-1.4	**-12.7**	-1.0	+1.1	-1.6
**Phenotypic**		LAMB1	+1.6	+1.3	-1.2	+1.3	-1.3	-2.0	-1.2
		SOX9	+2.59	+1.48	-1.32	**+6.79**	-1.65	-3.49	**+2.74**
	**MSC**	BGLAP	**+2.02**	-1.13	+1.95	**+3.51**	**-2.37**	-1.12	+1.58
		PPARG	**+27.62**	+9.90	+9.53	**+19.86**	-2.79	+1.24	-1.41
		ACAN	**-14.1**	-6.3	-2.5	**-16.2**	+2.1	-1.2	-1.2
		COL1A1	-1.1	+1.2	**-29.7**	-3.4	+1.3	**-27.3**	-2.9
		COL10A1	+1.1	**+60.4**	+2.2	**+61.9**	**+52.1**	+1.7	**+51.8**
**Matrix proteins**		COL2A1	-1.0	+49.2	-1.0	**+15.4**	+32.2	-1.0	**+8.1**
		COL3A1	**+3.6**	**+3.9**	-11.7	-2.2	+1.1	**-42.9**	-7.4
		eln	-1.4	**+12.9**	-3.0	-2.4	**+17.4**	-20.8	-1.8
		HAS1	**-7.3**	+1.3	+1.0	+1.1	**11.4**	+7.5	+9.3
	**Aggrecanases**	ADAMTS4	**+6.4**	+4.9	+2.2	**+26.4**	-1.3	-2.9	**+4.2**
		ADAMTS5	+1.1	-2.5	+3.2	+5.1	-2.6	+2.9	+5.2
**Matrix enzyme**		MMP1	**+369.3**	+9.7	**+86039.1**	**+28040.4**	-3.2	**+231.6**	**+78.2**
	**MMPs**	MMP13	**+2637.8**	**+3056.0**	+1085.0	**+15582.2**	+1.1	-2.5	**+6.0**
		MMP14	**+4.5**	**+2.2**	**+6.2**	**+6.8**	-2.1	+1.3	+1.5
		MMP2	**+3.8**	+1.8	**+4.7**	**+4.0**	-2.1	+1.2	+1.0
		MMP3	+36.1	+184.4	**+203663.7**	**+1973.3**	+11.7	**+12436.5**	+120.9
		MMP9	+22.2	**+5.9**	**+19.1**	**+3.2**	-3.8	-1.1	-7.3
		TIMP1	**+4.99**	+1.77	**+5.90**	**+9.12**	-2.60	+1.26	+1.79
**Anti-catabolic**		TIMP2	+1.46	-1.10	+1.26	+1.66	-1.64	-1.09	+1.14
		TIMP3	-1.00	-1.00	-1.00	+3.68	-1.00	-1.00	+3.08
		TGFB1	+1.82	+1.92	**+2.01**	**+3.96**	+1.06	+1.07	**+2.11**
		TGFB2	+1.43	-3.34	**-4.91**	-1.80	**-4.86**	**-6.78**	-2.49
**Growth factors**	**TGFB**	TGFB3	**+8.31**	+1.36	+3.08	**+5.27**	**-6.23**	**-2.97**	-1.58
		TGFBR1	-4.52	-4.61	-1.03	+1.05	-1.00	**+4.19**	**+4.83**
		TGFBR2	+1.03	+1.65	+1.25	+1.17	1.59	+1.18	+1.11
		CTGF	**-8.9**	**-3.9**	**-76.5**	**-9.6**	**+2.4**	**-8.2**	-1.0
		EGF	**-3.3**	-7.1	-1.1	-1.3	-2.1	+1.5	+2.4
		FGF1	-1.6	-1.5	+1.6	**+9.2**	+1.1	+2.4	**+14.1**
	**General**	IGF1	**+169.7**	+104.0	-1.0	+87.4	-1.6	-1.0	-1.9
		PDGFA	-1.63	-1.57	-1.39	-1.06	+1.02	+1.15	+1.46
		WISP1	+3.27	+2.62	-2.44	+1.90	-1.35	**-8.23**	-1.75
		IL1B	+22.7	+15.1	**+9911.6**	**+444.5**	-2.1	+310.6	+14.7
		TNF	+1.01	+1.66	**+90.93**	+5.33	+1.56	**+72.86**	+3.32
**Inflammatory**		CASP3	+1.3	-1.0	-1.2	+1.2	-1.3	-1.8	-1.1
		BDNF	-12.9	-4.7	-1.3	-6.3	-1.2	+3.1	+2.0
		NGF	-4.0	-2.7	+1.4	**-4.4**	-1.6	+1.6	-1.1
		TAC4	-1.08	+1.03	+4.22	+1.28	+1.09	**+4.33**	+1.36

#### Matrix-associated protein genes

B showed a significant increase in *COL3A1 *gene expression and decreases in *ACAN *and *HAS1 *expression relative to Day 0 (Table 2; Figure 1A). C demonstrated significant increases *COL10A1 *and *COL3A1 *relative to Day 0. However NCA showed significant decreases in *COL1A1 *and *COL3A1 *gene expression relative to Day 0 and B. NCT significantly increased *COL2A1 *and *COL10A1 *gene expression relative to Day 0 and B (Figure 1A).

#### Catabolic/anti-catabolic genes

A general up-regulation in the expression of catabolic enzymes was observed for all media groups post culture. B showed significant increases in *ADAMTS4, MMP1, -13, -14 *and *-2 *relative to Day 0 (Table 2; Figure 1B). C demonstrated significant increases in *MMP13, -14*, and *-9 *relative to Day 0. NCA showed significant increases in *MMP14 *and *-9 *relative to Day 0, and *MMP1,-2*, and *-3 *relative to Day 0 and B. NCT demonstrated significant increases in *MMP14, -3 *and *-9 *relative to Day 0 and *ADAMTS4, MMP1 *and *-13 *relative to Day 0 and B. Of the anti-catabolic proteins assessed, only *TIMP1 *showed significant increases in gene expression for B, NCA, and NCT relative to Day 0 (Table 2; Figure 1B).

#### Growth factor genes

In B, relative to Day 0, gene expression of *TGFB3 *and *IGF *was significantly up regulated and *CTGF *and *EGF *were significant down regulated (Table 2 Figure 1C). A significant decrease in *CTGF *expression relative to Day 0 and B was observed for C. NCA showed a significant increase in *TGFB1 *expression relative to Day 0 and decreases in *TGFB2 *and *CTGF *expression relative to Day 0 and B. NCT demonstrated significant increases in a number of growth factors; *TGFB1 *relative to Day 0 and B, and *TGFB3 *relative to Day 0 only including a significant down regulation in *CTGF *expression relative to Day 0.

#### Inflammatory/pain genes

B showed a significant decrease in *BDNF *expression relative to Day 0 (Table 2). C demonstrated no significant changes relative to Day 0 or B. In NCA a significant up-regulation of *IL-1B *relative to Day 0 was observed and *TNFα *was increased relative to both Day 0 and B. NCT also demonstrated a significant up-regulation of *IL1B*, however unlike NCA it down-regulated *NGF *relative to Day 0.

### Cell viability

The majority of cells appeared alive and healthy (stained green) with very few dead cells (stained red) after three weeks culture (data not shown). There are no differences in cell viability between media groups. This suggests that despite all media conditions demonstrating some catabolic/inflammatory effects they did not induce cell death.

### GAG and DNA content

**Figure 2:**
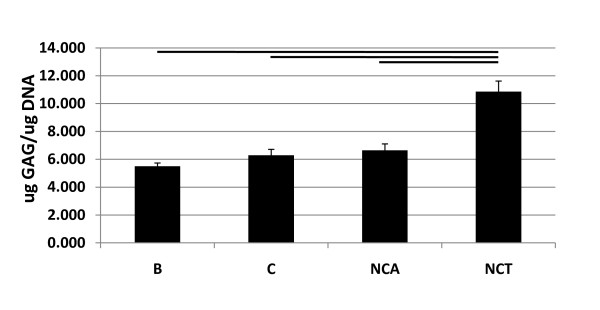
**Quantification of proteoglycan in MSC pellets**. Di-methyl methylene Blue (DMMB) analysis was used to assess the amount of Glycosaminoglycan (GAG) associated with the human mesenchymal stem cell (MSC) pellet 21 days post culture with four media groups; B = Basal, C = Chondrogenic, NCA = notochordal NP cells in alginate; NCT = notochordal NP cells in tissue normalized to DNA content using the Picogreen assay (μgGAG/μgDNA). Bar indicates significance, *P *< 0.05. Significantly more GAG was associated with the pellets of the NCT group in comparison to all other media groups.

GAG in the pellet (normalized to DNA content): A measurable amount of GAG was observed in the cells pellets of all media groups; however a significantly greater amount of GAG was demonstrated in the NCT relative to B, C and NCA (10.99 μg +/- 0.76 compared to 5.50 μg +/- 0.23, 6.28 μg +/- 0.43 and 6.64 μg +/- 0.46 (μg GAG/μg DNA) respectively) (Figure 2). GAG released to media (normalized to DNA content): No significant differences in GAG released to media were observed between media groups (data not shown). No significant differences in DNA content were observed between B, C and NCA compared to the NCT group (0.162 μg +/- 0.009, 0.149 μg +/- 0.009, 0.172 μg +/- 0.008 compared to 0.134 μg +/- 0.009 respectively) (Additional file 4 Figure S4).

### Histology of pellets

**Figure 3:**
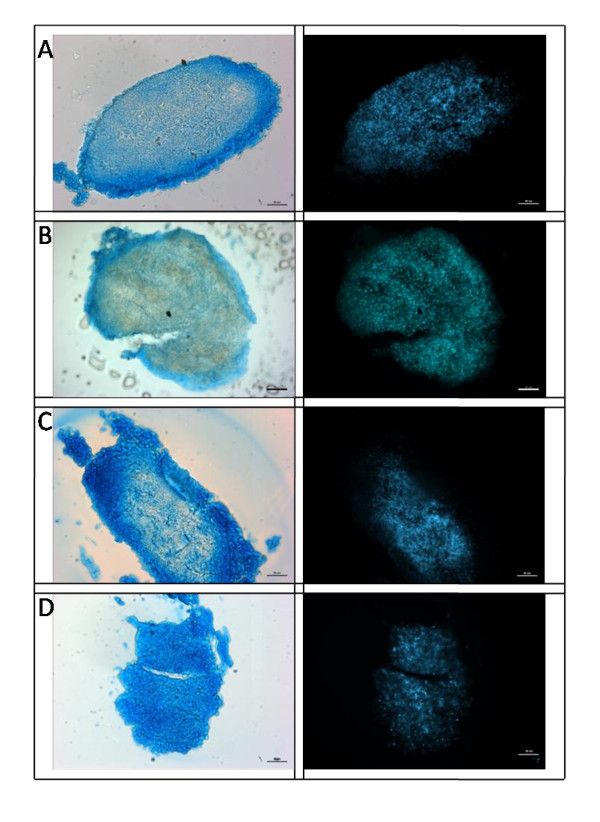
**Histology of MSC pellets 3 weeks post culture**. Human mesenchymal stem cell (MSC) pellets were sectioned at 20 μm and stained with Alcian Blue (for GAG) and 4',6-diamidino-2-phenylindole (DAPI) (cell nuclei) after 21 days culture with four media groups; either B = Basal, C = Chondrogenic, NCA = notochordal NP cells in alginate; NCT = notochordal NP cells in tissue. (scale bar = 50 μm). GAG was observed for all media groups however NCT demonstrated the greater abundance of GAG throughout the whole pellet with fewer stained nuclei compared to other groups.

Alcian blue staining showed a general trend of light staining in the middle with stronger staining along the periphery of the pellet for B, C and NCA media groups; however, NCA demonstrated darker staining along the periphery (Figure 3). The NCT media group showed the most dramatic staining throughout, indicating that this media type produced the most proteoglycans. DAPI staining showed uniform staining throughout the pellet with fewer cells appearing along the periphery. C and NCA media groups were the most cellular with NCT being the least. This is consistent with picogreen assay data which demonstrated a greater DNA content for C and NCA compared to NCT (data not shown).

### Proteomics of media groups

To determine if the NCT and NCA conditioned media showed major differences in protein profiles compared to the unconditioned control media, and as a first approach to determine if porcine-specific protein factors could even be identified in the conditioned media, equal volumes of each medium were subjected to denaturing SDS-PAGE and the gel was stained with coomassie blue to visualize proteins. Several proteins were identified in both regions of the conditioned media samples that were not identified in the control sample. However, only three proteins were identified by more than one peptide whose amino acid sequences were unambiguously porcine in origin (Table 3). The "." in each peptide sequence indicates the site of tryptic cleavage and is preceded (at the amino-terminus) or followed (at the carboxyl-terminus) by the amino acid in the porcine sequence. Also indicated are the pre-processed predicated molecular masses of the protein precursors. BLAST searches were performed on each peptide and if the bovine protein ortholog had an identical sequence (allowing for isobaric exchanges between leucine and isoleucine) it is so noted. Values of multiple parameters of the proteomics SEQUEST search for each peptide are listed and include the cross correlation score (XCorr), the charge state (*z*), the delta correlation score of the next unique peptide sequence (Unique ΔCn), and the measured mass difference of the precursor compared to its theoretical mass in parts per million *m/z *(Δppm). Also noted are the number of times each peptide was identified and the number of entries in the non-redundant protein database reported by SEQUEST with the exact amino acid sequence of the peptide. § indicates a redundancy was uncovered in bovine by BLAST searching. "M*" denotes an oxidized methionine residue. All three of the porcine-specific identified proteins originated uniquely from the NCA sample: clusterin found in the 37 kDa range and tenascin and alpha-2-macroglobulin found in the 140 kDa range. Table 3 lists the peptides identified from these three proteins in each sample and details their relevant proteomic metrics.

**Table 3:** Porcine-specific proteins/peptides identified via proteomic analysis of NCA

NCA region	Porcine Protein and Peptides Identified	Identical in Bovine Ortholog	XCorr	*z*	Unique ΔCn	Times Identified	Redundancy in Database
140 kDa	**alpha-2-macroglobulin precursor (163 kDa)**						
	K.IKEEGTEVELTGK.G	No	4.311	2	0.359	2	0
	R.SSGSLLNNAIK.G	No	3.07	2	0.343	2	5
	R.TPQIITILEK.A	No	2.95	2	0.324	2	0
	R.KYSNPSTCFGGESQAICEK.F	No	2.646	3	0.129	1	0
	R.QEFEM*KLEVEAK.I	Yes	1.823	3	0.079	1	1§
	K.YGAATFTR.T	No	1.716	2	0.17	1	10
140 kDa	**tenascin precursor (191 kDa)**						
	K.ATLTGLRPGTEYGIGVSAVK.G	No	4.872	3	0.564	1	0
	R.LNYGLPSGQPVEVQLPR.N	No	4.721	3	0.51	2	0
	R.GLEPGQEYTILLTAEK.G	No	3.424	2	0.388	2	0
	R.VATYLPTPEGLK.F	Yes	2.706	2	0.34	2	1§
	K.ESSLTLLWR.T	No	2.582	2	0.308	2	0
	R.VPGDQTSTTIR.E	No	2.418	2	0.301	2	4
37 kDa	**clusterin precursor (52 kDa)**						
	R.ASNIM*DELFQDR.F	No	4.341	2	0.449	1	0
	R.KSLLSSLEEAKK.K	No	3.739	3	0.231	2	0
	K.TLIEQSNEERK.S	No	3.135	2	0.232	2	0
	R.QQSHVM*DIM*EDSFNR.A	No	3.08	3	0.235	2	0
	K.AISDKELQEM*STEGSK.Y	Yes	3.051	3	0.333	2	1§
	K.TLIEQSNEER.K	No	2.692	2	0.326	3	0

## Discussion

Human MSCs are a potential cell source for regeneration of the degenerated human IVD yet the appropriate method for pre-differentiation remains unclear. This study differentiated human MSCs for 21 days, using two notochordal cell media conditions as well as chondrogenic and basal media groups. The lack of clear NP phenotypic markers led to examination of a number of outcome measures including use of gene profiling with a custom PCR array of 42 genes associated with a healthy NP phenotype, an important innovation of this study, as well as assessments of GAG, histology, and cell viability. Culture of human MSCs in NCT stimulated anabolic changes most similar to a healthy NP phenotype rather than a chondrogenic phenotype with increased proteoglycan, while NCA conditioning resulted in significant down-regulation of fibrotic genes and minimal effects on the hypertrophic gene *COLX*. Chondrogenic and basal groups demonstrated many similarities in gene expression compared to Day 0 (pre-culture) conditions, suggesting that micromass culture under hypoxic conditions produces a similar gene profile as MSCs cultured with Chondrogenic media.

NCT conditioning of MSCs resulted in up-regulation of *SOX9*, *COL2*, and *TGFB3 *that could be associated with a healthy NP phenotype [10,27,28]. This was corroborated at the protein level with a significant increase in GAG associated with the cell pellet as shown by the DMMB assay and Alcian blue staining despite a decrease in *ACAN *at the gene level. The increase in GAGB observed for NCT relative to other media groups suggests that the cell phenotype induced with NCT was more closely an NP than chondrocytic phenotype. NCT also demonstrated an increase in matrix enzymes and *IL-1B*. However, because significant increases in most anabolic genes and GAG were also observed, it is possible that the catabolic effects induced by NCT are associated with remodeling during differentiation rather than a catabolic cell phenotype [41].

NCA had an anti-fibrotic effect on MSC differentiation with significant down-regulation of *COL1A1 *and *COL3A1*. Whilst significant increases were observed in *COLX *for both NCT and C, for NCA minimal changes in the hypertrophic marker *COLX *were noted. A common problem of *in vitro *induced chondrogenesis is hypertrophic differentiation of MSCS with increased expression of Collagen × [29,42]. Hypertrophic differentiation and calcification has also been shown during intervertebral disc degeneration [43]. Minimal changes in *COLX *expression suggests that NC cells in alginate alone may produce soluble factors capable of limiting or preventing hypertrophy and inhibiting synthesis of certain fibrous proteins. Unlike NCT, NCA had little impact on anabolic gene expression however accumulation of GAG was observed in MSCs after 21 days. These results are in contrast to a previous study that used conditioned media from NCs in alginate constructs to treat human MSCs for seven days and found increases in expression of matrix proteins [32]. We can speculate that differences in gene expression may be due to the different time courses of the studies (7 days versus 21 days) or the differences in methods of CM generation.

A preliminary proteomics study demonstrated that NCA media conditions contained porcine alpha-2-macroglobulin, clusterin and tenascin. Intriguingly, these proteins have been implicated as cytoactive proteins that could be involved in reducing fibrous collagens, limiting matrix degradation or hypertrophy. Alpha-2-macroglobulin is an endoproteinase inhibitor present in blood and joint fluid which functions as a substrate for matrix enzymes such as ADAMTS-4 and -5 and inhibits their activity [44]. Clusterin is known to be a multifunctional, secreted glycoprotein expressed in diverse locations, implicated in regulating complement activation and cell death in injured and degenerating tissues, and may have a cytoprotective effect on chondrocytes including NP cells [45,46]. Tenascin is an extracellular matrix glycoprotein known to be abundant in the annulus of young IVDs and localized pericellularly in degenerated IVDs, and possibly could have a role in fibronectin - disc cell interactions [47]. The biological roles of these proteins were not tested in this study, therefore, their effects are speculative and require further validation to confirm such roles.

Effects observed for C were consistent with the chondrocyte cell phenotype (a trend of increasing *SOX9 *and *COL2 *expression) at the gene and also at the protein level with GAG detected in the cell pellet. Results for B showed many similarities to that of C including the presence of GAG in the cell pellet. The only principal differences were a lack of *COL2 *expression and up-regulation of the phenotypic marker *GPC1*, growth factor *TGFB3*, and anti-catabolic protein *TIMP1*. These changes were unexpected as B was a control group. This suggests that the initial dose of TGFβ-3 for 24 hours followed by 3D culture/hypoxia for three weeks was sufficient to differentiate MSCs toward a chondrogenic phenotype. As a consequence of these unexpected findings, relative gene expression was normalized to Day 0, undifferentiated human MSCs, rather than B when examining the effects of NCA and NCT. Consequently, certain genes (that is, catabolic) were expressed at extremely low levels at Day 0 and relative expression levels are at very high orders of magnitude for all groups.

Until very recently no definitive markers of the IVD or NP cell phenotype were available, therefore markers of the chondrocyte phenotype were often used to assess MSC differentiation (for example, SOX9) [9,27]. Microarray analysis of rat, bovine and canine IVD tissue has identified several candidate phenotypic markers such as Glypican, Biglycan, Keratin 19 and Laminin B1 [22,23]. However, studies have also shown that species differences and degree of degeneration can influence the magnitude of expression of these genes, questioning their suitability as IVD/NP phenotypic markers [24,25]. In this study, little change at the gene expression level was observed for these markers. Optimal NP phenotypic markers are a moving target as research continues to advance, and recent studies identified up to 12 NP positive and 36 negative marker genes using microarray analysis of human IVD cells, a subset of which were then examined in differentiated human MSCs [26]. Future studies, therefore, require investigation of such markers to accurately assess differentiation of MSCs toward an NP phenotype.

The enhanced production of GAG in the NCT group suggests that NCs in their native tissue environment were able to differentiate MSCs toward an NP phenotype. At the gene level a decrease in aggrecan expression was noted however as protein data (GAG in pellets and histology) confirmed the presence of proteoglycan it is speculated that this effect may correspond to a negative feedback loop; as aggrecan has been made the cells down-regulate its gene expression, or alternately to the accumulation of GAG without production of aggrecan core protein. The increased GAG content is likely associated with greater total accumulation of GAG (as observed histologically with no differences in cell viability per group) as well as increased GAG per cell. The notochordal rich ECM is likely to influence the proteins present in the notochordal conditioned media from tissue and could be responsible for the observed effects on MSCs for this media group. Indeed the observed effects are likely a consequence of either soluble factors produced by NCs only when they are situated in their native matrix or factors derived from the matrix itself (for example, matricellular proteins). Although the SDS-PAGE and proteomic analysis of the NCT conditioned did not reveal observable differences in specific growth and differentiation factors, due largely to the masking effect of the bovine serum albumin in the samples, previous studies suggest many candidates. For example, the matricellular protein CTGF (connective tissue growth factor) has been implicated as an anabolic factor responsible for the effects mediated by NCCM on IVD biosynthesis [48]. In this study, we observed significant down-regulation of CTGF at the gene level for all media groups. We suggest that this decrease in CTGF may represent a negative feed-back mechanism in which CTGF may have been synthesized at the beginning of culture or been present at sufficient levels in the CM.

This study used an *in vitro *micromass culture system with human MSCs and porcine notochordal derived conditioned media for differentiation toward a healthy NP phenotype. This cross-species comparison was justified as human BM-MSCs are currently the most clinically relevant cell source for disc repair and porcine notochordal cells unlike human notochordal cells are readily available; however, it cannot be ruled out that species differences may have had an impact on the results obtained and is, therefore, a limitation of the study. Our goal is to use the NC conditioned media to identify proteins as therapeutic agents and not to ultimately use porcine tissue to generate the therapeutic agent. Thus, this is solely an experimental model and there would not be a cross-species concern if this approach were ultimately used clinically. Proteoglycan measurements using both the DMMB assay and histological analyses using Alcian blue demonstrated similar trends adding confidence to both measurements; however, the presence of guanidine thiocyanate in the cell pellet lysis solution may interfere with the DMMB assay and could have an effect on total GAG content [49]. The relatively small sample size can also be considered a limitation however trends were consistent between specimens and only significant changes were discussed here. NCA and NCT demonstrated differing effects on MSC differentiation at the gene and protein level and differences may be accounted for by the native cell-matrix interactions present in NCT compared to NCA with cells cultured alone. Alternatively cell extraction for the NCA group may have affected notochordal cell phenotype therefore gene profiling pre and post conditioning would be a necessary next step to determine this. NCA and NCT also demonstrated differences with regard to proteomic analysis with proteins identified in NCA only. This could be explained by the presence of BSA in the media masking larger matricellular proteins derived from native notochordal tissue and also the different cell-matrix environment as mentioned above. These lines of inquiry as well as proteomic analysis of albumin-free conditioned media will be the subject of future studies.

## Conclusions

Using a custom PCR array of 42 genes associated with the healthy NP cell phenotype we have shown that CM derived from NCs had diverse effects on MSC differentiation toward a NP phenotype and this was dependent on the conditions in which the CM was generated. In their native IVD matrix NCs enhanced MSC differentiation toward an NP phenotype with increased production of GAG whilst CM derived from NCs alone cultured in alginate inhibited fibrotic genes and induced minimal effects on hypertrophic gene expression compared to standard chondrogenic media containing TGFβ-3. This was confirmed by histology and analysis of GAG in pellets. Likely candidates for the observed effects include anabolic matricellular proteins derived from the NC matrix itself. However, CM from NCs alone in alginate warrants further investigation due to the inhibitory effects observed on fibrotic genes and minimal effects on hypertrophic matrix proteins, of which clusterin and tenascin are possible candidate proteins identified in this study which require further validation. The development of an optimal method to pre-condition MSCs for injection into a degenerated IVD depends on our ability to successfully combine multiple factors. In addition to correctly formulated media, appropriate culture conditions will include proper MSC microenvironment (cell-cell/cell-matrix), and oxygen tension and mechanical stimulus. Once this has been realized, a therapy in which MSCs can restore the health of a degenerated IVD may be possible.

## Abbreviations

ADAMTSs: A distintegrin and metalloproteinase with thrombospondin motifs; AF: Annulus fibrosus; B: basal; C: Chondrogenic; CTGF: connective tissue growth factor; CM: conditioned media; DAPI: 4',6-diamidino-2-phenylindole; DMB: Di-methyl methylene Blue; ETH-2: ethidium Homodimer-2; GAG: glycosaminoglycan; IL-1 β: interleukin-1 beta; ITS: Insulin-Transferrin-Selenium; IVD: intervertebral disc; LC-MS/MS: Liquid Chromatography tandem mass spectrometry; MSCs: mesenchymal stem cells; MMPs: matrix metalloproteinases; NCA: notochordal cells in alginate; NCCM: conditioned medium derived from NCs; NCs: notochordal cells; NCT: notochordal cells in tissue; NP: nucleus pulposus; SDS-PAGE: Sodium Dodecyl Sulfate-Polyacrylamide Gel Electrophoresis; TGFβ-3: transforming growth factor beta 3; TNFα: tumor necrosis factor alpha.

## Competing interests

The authors declare that they have no competing interests.

## Authors' contributions

DP was involved in the study design, performed all experimental work, data analysis and interpretation, and drafted the manuscript. RMS participated in the study design, data interpretation, and helped to draft the manuscript. RDA contributed to the study design, experimental work, data analysis and interpretation. BB performed the SDS-PAGE and proteomic assessment of media groups, data analysis and write-up and helped with data interpretation. KEG participated in the study design, experimental work and data analysis. JCI secured funding, contributed to the study design, organized and executed the study and helped with data analysis and interpretation including drafting the manuscript. All authors read and approved the manuscript.

## Supplementary Material

Additional file 1 **Table S1**. The 42 genes associated with NP phenotype assessed in human MSCs treated with Basal, Chondrogenic, media from Notochordal NP cells in alginate and Notochordal NP cells in tissue using custom qRT-PCR array (SYBR green). Click here for file

Additional file 2 **Table S2**. The complete gene names of the 42 genes associated with NP phenotype assessed. Click here for file

Additional file 3 **Figure S3**. Coomassie-stained SDS-PAGE gel of equal volumes of control (or Basal medium prior to conditioning), NCA and NCT medias. Molecular weight standards are in the first lane and their values are in kDa. Asterisks denote the approximately 140 kDa and approximately 37 kDa regions that were cut from each lane and subjected to proteomic analysis. Click here for file

Additional file 4 **Figure S4**. DNA content in MSC cell pellets 21 days after treatment with Basal, Chondrogenic, media from Notochordal NP cells in alginate and Notochordal NP cells in tissue assessed using the Picogreen Assay (μg DNA per pellet). Click here for file
