# Supplementary material for: Notochordal conditioned media from tissue increases proteoglycan accumulation and promotes a healthy nucleus pulposus phenotype in human mesenchymal stem cells
Source: Arthritis Res Ther. 2011 May 31;13(3):R81. doi: 10.1186/ar3344 (PMC3218891; doi:10.1186/ar3344)
Supplement: Additional file 1 — Table S1. The 42 genes associated with NP phenotype assessed in human MSCs treated with Basal, Chondrogenic, media from Notochordal NP cells in alginate and Notochordal NP cells in tissue using custom qRT-PCR array (SYBR green). [file ar3344-S1.DOCX]

**Additional file 1, Table S1**

| **Group** | | **Genes** |
| --- | --- | --- |
| **Phenotypic-related markers** | **IVD** | SOX9, BGN, KRT19, LAMB1, GPC1 |
|  | **MSCs** | PPARG, BGLAP |
| **Growth factors** | **TGFβ family** | TGFβ1, TGFβ2, TGFβ3, TGFβR1, TGFβR2 |
|  | **General** | CTGF, EGF, FGF1, IGF1, PDGFA, WISP |
| **Matrix-related proteins** | | ACAN, COL2A1, COL1A1, COL10A1, COL3A1, ELN, HAS1 |
| **Catabolic-related enzymes** | **Aggrecanases** | ADAMTS 4, ADAMTS 5 |
|  | **Matrix-metalloproteinases** | MMP1, MMP13, MMP14, MMP2, MMP3, MMP9 |
| **Anti-catabolic-related proteins** | | TIMP1, TIMP2, TIMP3 |
| **Inflammatory/pain proteins** | | IL-1B, TNFA, CAS3, BDNF, NGF,TAC4 |
