# Supplementary material for: Notochordal conditioned media from tissue increases proteoglycan accumulation and promotes a healthy nucleus pulposus phenotype in human mesenchymal stem cells
Source: Arthritis Res Ther. 2011 May 31;13(3):R81. doi: 10.1186/ar3344 (PMC3218891; doi:10.1186/ar3344)
Supplement: Additional file 2 — Table S2. The complete gene names of the 42 genes associated with NP phenotype assessed. [file ar3344-S2.DOCX]

**Additional file 2, Table S2**

| **Abbreviation** | **Full Gene Name** |
| --- | --- |
| **SOX9** | Transcription factor SOX-9 |
| **BGN** | Biglycan |
| **COL3A1** | Collagen type 3 alpha 1 |
| **KRT19** | Keratin 19 |
| **LAMB1** | Laminin subunit beta-1 |
| **GPC1** | Glypican |
| **PPARG** | Peroxisome proliferator-activated receptor gamma |
| **BGLAP** | Osteocalcin |
| **TGFβ1** | Transforming growth factor beta 1 |
| **TGFβ2** | Transforming growth factor beta 2 |
| **TGFβ3** | Transforming growth factor beta 3 |
| **TGFβR1** | Transforming growth factor beta receptor 1 |
| **TGFβR2** | Transforming growth factor beta receptor 2 |
| **CTGF** | Connective tissue growth factor |
| **EGF** | Epidermal growth factor |
| **FGF1** | Fibroblast (heparin-binding) growth factor 1 |
| **IGF1** | Insulin-like growth factor 1 |
| **PDGFA** | Platelet-derived growth factor subunit A |
| **WISP** | WNT1-inducible-signaling pathway protein 1 |
| **ACAN** | Aggrecan |
| **COL2A1** | Collagen type 2 alpha 1 |
| **COL1A1** | Collagen type 1 alpha 1 |
| **COL10A1** | Collagen type 10 alpha 1 |
| **ELN** | Elastin |
| **HAS1** | Hyaluronan synthase 1 |
| **ADAMTS 4** | A disintegrin and metalloproteinase with thrombospondin motifs 4 |
| **ADAMTS 5** | A disintegrin and metalloproteinase with thrombospondin motifs 5 |
| **MMP1** | Matrix-metalloproteinase 1 |
| **MMP13** | Matrix-metalloproteinase 13 |
| **MMP14** | Matrix-metalloproteinase 14 |
| **MMP2** | Matrix-metalloproteinase 2 |
| **MMP3** | Matrix-metalloproteinase 3 |
| **MMP9** | Matrix-metalloproteinase 9 |
| **TIMP1** | Tissue inhibitor metallopeptidase 1 |
| **TIMP2** | Tissue inhibitor metallopeptidase 2 |
| **TIMP3** | Tissue inhibitor metallopeptidase 3 |
| **IL-1B** | Interleukin 1 beta |
| **TNFA** | **tumor necrosis factor-alpha** |
| **CAS3** | Caspase 3 |
| **BDNF** | Brain-derived neurotrophic factor |
| **NGF** | Nerve growth factor |
| **TAC4** | Tachykinin 4, Substance P |
