# Supplementary figures and images for: Notochordal conditioned media from tissue increases proteoglycan accumulation and promotes a healthy nucleus pulposus phenotype in human mesenchymal stem cells
Source: Arthritis Res Ther. 2011 May 31;13(3):R81. doi: 10.1186/ar3344 (PMC3218891; doi:10.1186/ar3344)

**Additional file 3, Figure S3**


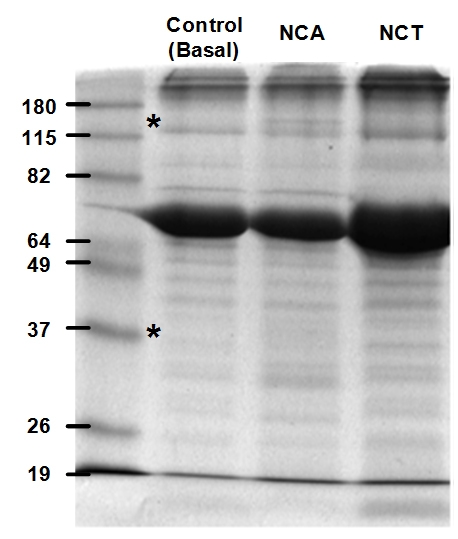

Supplement: Additional file 3 — Figure S3. Coomassie-stained SDS-PAGE gel of equal volumes of control (or Basal medium prior to conditioning), NCA and NCT medias. Molecular weight standards are in the first lane and their values are in kDa. Asterisks denote the approximately 140 kDa and approximately 37 kDa regions that were cut from each lane and subjected to proteomic analysis. [file ar3344-S3.DOCX]
