# Supplementary material for: Notochordal conditioned media from tissue increases proteoglycan accumulation and promotes a healthy nucleus pulposus phenotype in human mesenchymal stem cells
Source: Arthritis Res Ther. 2011 May 31;13(3):R81. doi: 10.1186/ar3344 (PMC3218891; doi:10.1186/ar3344)
Supplement: Additional file 4 — Figure S4. DNA content in MSC cell pellets 21 days after treatment with Basal, Chondrogenic, media from Notochordal NP cells in alginate and Notochordal NP cells in tissue assessed using the Picogreen Assay (μg DNA per pellet). [file ar3344-S4.DOCX]

**Additional file 4, Figure S4**
